# Theranostic SPECT reconstruction for improved resolution: application to radionuclide therapy dosimetry

**DOI:** 10.1186/s40658-021-00362-x

**Published:** 2021-02-17

**Authors:** H. Marquis, D. Deidda, A. Gillman, K. P. Willowson, Y. Gholami, T. Hioki, E. Eslick, K. Thielemans, D. L. Bailey

**Affiliations:** 1 Sydney Vital Translational Cancer Research Centre, Sydney, Australia; 2 Institute of Medical Physics, University of Sydney, Sydney, Australia; 3 National Physical Laboratory, Teddington, UK; 4 Australian e-Health Research Centre, CSIRO, Brisbane, Australia; 5 Department of Nuclear Medicine, Royal North Shore Hospital, Sydney, Australia; 6 Faculty of Medicine and Health, University of Sydney, Sydney, Australia; 7 Institute of Nuclear Medicine, University College London, London, UK

**Keywords:** Radionuclide therapy, Tumour dosimetry, Theranostics, Partial volume correction

## Abstract

**Background:**

SPECT-derived dose estimates in tissues of diameter less than 3× system resolution are subject to significant losses due to the limited spatial resolution of the gamma camera. Incorporating resolution modelling (RM) into the SPECT reconstruction has been proposed as a possible solution; however, the images produced are prone to noise amplification and Gibbs artefacts. We propose a novel approach to SPECT reconstruction in a theranostic setting, which we term SPECTRE (single photon emission computed theranostic reconstruction); using a diagnostic PET image, with its superior resolution, to guide the SPECT reconstruction of the therapeutic equivalent. This report demonstrates a proof in principle of this approach.

**Methods:**

We have employed the hybrid kernelised expectation maximisation (HKEM) algorithm implemented in STIR, with the aim of producing SPECT images with PET-equivalent resolution. We demonstrate its application in both a dual ^68^Ga/^177^Lu IEC phantom study and a clinical example using ^64^Cu/^67^Cu.

**Results:**

SPECTRE is shown to produce images comparable in accuracy and recovery to PET with minimal introduction of artefacts and amplification of noise.

**Conclusion:**

The SPECTRE approach to image reconstruction shows improved quantitative accuracy with a reduction in noise amplification. SPECTRE shows great promise as a method of improving SPECT radioactivity concentrations, directly leading to more accurate dosimetry estimates in small structures and target lesions. Further investigation and optimisation of the algorithm parameters is needed before this reconstruction method can be utilised in a clinical setting.

## Background

Nuclear medicine functional imaging with positron emission tomography (PET) and single photon emission computed tomography (SPECT) are used extensively for the diagnosis of disease, planning/staging of therapy, radionuclide therapy (RNT) dosimetry calculations and monitoring of response to treatment. More recently, the term “theranostic” has been introduced in nuclear medicine to describe a single molecule, peptide or other targeted agent that is used for both diagnostic imaging and radionuclide therapy labelled with different radionuclides.^1^ This concept of managing patients is now, more generally, referred to as a “theranostic paradigm”. Well-known examples include the use of radioiodine to treat thyroid cancer and other thyroid disorders, and the use of radiolabelled octreotate (or similar somatostatin receptor-targeting peptides) in the management of patients with neuroendocrine cancer.

One major limitation that nuclear medicine imaging faces, however, is relatively poor spatial resolution giving rise to significant partial volume effects (PVEs) which result in significant underestimation of radioactivity concentration in volumes less than approximately 2–3 times the spatial resolution (full width at half-maximum, FWHM) of the imaging system [2]. It has been demonstrated that the spatial resolution in SPECT imaging with medium-energy gamma photons (~ 200 keV) associated with therapeutic radionuclides such as lutetium-177 (^177^Lu) and copper-67 (^67^Cu) of approximately 20 mm FWHM severely limits the recovery of accurate radioactivity concentrations in vivo due to the PVE [3] and hence leads to a large underestimation in radiation dose delivered to small structures in peptide receptor radionuclide therapy (PRRT) such as metastatic foci and nodal disease. Estimating the dose delivered to small lesions in RNT using conventional SPECT reconstruction methods is therefore compromised and thus partial volume correction (PVC) is essential for accurate RNT treatment planning, monitoring of therapeutic dose and establishing the relationship between RNT and treatment outcome, i.e. dose/response relationships.

### Partial volume correction in emission tomography

The aim of PVC is to mitigate the degradation in the reconstructed image due to the finite system resolution from a PET or SPECT acquisition, so as to recover the true radioactivity concentration in tissues of interest [4]. SPECT PVC can be performed during the reconstruction process by modelling the collimator-detector response, known as *resolution modelling* (RM) or via deconvolution algorithms applied to the images after reconstruction. Resolution modelling during the reconstruction process is reported to have superior noise characteristics compared to post-reconstruction deconvolution methods [4]. Both approaches to PVC can result in the production of Gibbs’ artefacts, corresponding to “ringing” at sharp edges due to missing high spatial frequency information [4, 5]. One solution is to incorporate prior knowledge into the reconstruction process, which improves the resolution while encoding additional information about the typical radioactivity distribution to reduce the presence of Gibbs artefacts. The introduction of a priori information into nuclear medicine image reconstruction has commonly used anatomical imaging modalities such as MRI and CT, where the assumption is that functional regions of tracer uptake are correlated to spatial regions with differing signals in the MRI or X-ray CT image [4, 6–9]. Guided image reconstruction imposes penalties on the reconstruction solution based on information coming from the prior image, so that the result is smooth in regions where uniformity is expected, and sharp across anatomical boundaries; thereby controlling noise and minimising the onset of ringing artefacts, while also correcting for PVEs [10–12]. Resolution modelling incorporating anatomical priors has been shown to improve quantification accuracy in small lesions [13].

Many reconstruction approaches and algorithms have been proposed to incorporate prior information from one modality to guide the reconstruction of tomographic emission data. These include separately reconstructing pre-defined segments of the image using an existing smoothness prior, such as the Gibbs smoothing prior [14]; the use of multi-modal similarity metrics, such as mutual information [15]; smoothing only across a subset of neighbours that are similar in the guiding modality [8]; and the kernelised expectation maximisation (KEM) method, reconstructing an image that is a linear combination of a set of basis-functions (determined by “kernels”) that are found based on the local structure in the image of the guiding modality [12, 16]. Each of these approaches have primarily been applied to multi-modality imaging that have expected correlations between radiotracer uptake and anatomical/morphological information, and thus make reasonably strong assumptions about the shared boundaries between the functional image and the guiding modality. This can result in the suppression of tracer uptake in small structures, such as small lesions or metastatic disease, by over-smoothing if the tracer uptake does not correspond to anatomical boundaries or the information present in the guiding modality [7, 17, 18]. A recent SPECT reconstruction algorithm that is commercially available is “xSPECT Bone” (Siemens Healthineers, Hoffman Estates, USA), an iterative conjugate gradient optimiser-reconstruction algorithm [19] for SPECT that incorporates anatomical information from CT image data into the SPECT reconstruction process, with the aim of producing high-resolution SPECT images [20]. This reconstruction algorithm is currently limited to few unique applications, such as technetium-99m (^99m^Tc) bone scanning.

A recent development involves the use of a hybrid kernel, incorporating high-resolution anatomical data from MRI or CT and the emission (PET or SPECT update image) to reconstruct PET or SPECT emission data. This algorithm, known as the hybrid kernelised expectation maximisation (HKEM) algorithm [21], is a generalisation of the KEM algorithm and uses both the guiding image (anatomical or functional) *and* the emission data, to iteratively reconstruct the PET or SPECT image. The HKEM approach to PET image reconstruction was initially developed to address the issue of suppression of features unique to the PET data, the idea being that a hybrid kernel could minimise PET-unique feature suppression in MR-guided PET KEM reconstructions. The hybrid kernel method was shown to better preserve features unique to the PET data compared to KEM [22]. Generally, the guiding modality used in these techniques is a structural modality, often MRI or CT. In the few cases previously explored where the guiding modality was functional, the application has used static reconstructions to guide dynamic frames within the same acquisition [12]. In many theranostics applications, radiopharmaceutical pairs are used to first perform a diagnostic PET scan for planning, followed by administration of the therapeutic equivalent, where dose monitoring is performed on the SPECT camera. These images show very similar functional distributions, but with vastly differing spatial resolution. We propose to use the theranostic paradigm and apply it to the image reconstruction process to mitigate the impact of the limited spatial resolution of SPECT imaging of many therapeutic radiopharmaceuticals (e.g. ^177^Lu, ^67^Cu). The aim is to utilise the superior spatial resolution of PET to improve the reconstructed spatial resolution of SPECT. We base the reconstruction algorithm on HKEM, as opposed to KEM, to allow differences in pharmacokinetics to manifest and to avoid over-regularising regions unique to the SPECT data. We refer to this as a theranostic image reconstruction (TIR) approach, which, when applied to the gamma camera data we call SPECTRE (single photon emission computed theranostic reconstruction). SPECT image reconstruction using PET as the guiding information has not previously been reported to the best of our knowledge. The aim of this paper is to demonstrate the proof in principle of SPECTRE in both quantitative phantom experiments with small target objects and in clinical examples.

## Methods

We will demonstrate our initial implementation of SPECTRE using an experimental phantom as well as a clinical in vivo theranostic application. Both examples use a PET scan as the guiding modality for the SPECT reconstruction of the therapeutic radionuclide. The phantom study uses a gallium-68 (^68^Ga) PET scan combined with a subsequent ^177^Lu SPECT scan of the test object, the IEC image quality phantom [23]. The clinical application uses a copper-64 (^64^Cu) PET scan of a somatostatin-receptor targeting ligand combined with a ^67^Cu SPECT scan of the therapeutic isotope using the same targeting ligand. The HKEM algorithm used in the SPECTRE reconstructions is implemented in the STIR (Software for Tomographic Image Reconstruction) software package^2^ [24] and is open source. The PET and SPECT kernels used in the SPECTRE implementation of the HKEM algorithm can be represented mathematically as

$$ {k}_{p\  fj}={k}_p\left({v}_f,{v}_j\right)=\mathit{\exp}\left(-\frac{{\left\Vert {v}_f-{v}_j\right\Vert}^2}{2{\sigma}_p^2}\right)\mathit{\exp}\left(-\frac{{\left\Vert {x}_f-{x}_j\right\Vert}^2}{2{\sigma}_{dp}^2}\right) $$

and

$$ {k}_{s\  fj}^{(n)}={k}_s\left({z}_f^{(n)},{z}_j^{(n)}\right)=\mathit{\exp}\left(-\frac{{\left\Vert {z}_f^{(n)}-{z}_j^{(n)}\right\Vert}^2}{2{\sigma}_s^2}\right)\mathit{\exp}\left(-\frac{{\left\Vert {x}_f-{x}_j\right\Vert}^2}{2{\sigma}_{ds}^2}\right) $$

where *k*_*p*_ and *k*_*s*_ represent the kernels derived from the PET prior and the SPECT iterative update respectively. The kernels are defined such that they map the similarity between the local voxel index, *f*, and the “supporting” voxel index, *j,* allowing arbitrary associations to “support” the reconstructed image (i.e. make more similar). The Gaussian-weighted strength of the support from the neighbourhood is defined by the PET (*v*_*f*_ and *v*_*j*_) and SPECT (*z*_*f*_ and *z*_*j*_) intensity, i.e. more similar PET and SPECT locations provide more support, and the voxel location (*x*_*f*_ and *x*_*j*_), i.e. closer locations provide more support. Parameters σ_p_ and σ_s_ control the degree of edge preservation coming from the PET prior and the SPECT update image respectively, and σ_dp_ and σ_ds_ are scaling parameters that control the degree of edge preservation for σ_p_ and σ_s_ in terms of Euclidean distances (mm), i.e. they are weights assigned to the voxels that lie within the kernel search window; smaller σ_dp_ and σ_ds_ values result in a greater significance assigned to voxels closer to the centre voxel in the kernel search window. These kernels are then incorporated into a maximum likelihood expectation maximisation reconstruction using

$$ {\alpha}_f^{\left(n+1\right)}=\frac{\alpha_f^{(n)}}{\sum_j{k}_{p\  fj}{k}_{s\  fj}^{(n)}{\sum}_i{p}_{ij}}{\sum}_j{k}_{p\  fj}{k}_{s\  fj}^{(n)}{\sum}_i{p}_{ij}\frac{1}{\sum_l{p}_{il}{\sum}_{f`}{k}_{p\ f`l}{k}_{s\ f`l}^{(n)}{\alpha}_{f`}^{(n)}+{s}_i} $$

where ${\alpha}_f^{(n)}$ is the coefficient image at each location, *f*, at the *n*-th iteration, *f*′ is the inner-loop index akin to *f* but prior to forward projection, *p*_*ij*_ is the system matrix, and *s*_*i*_ is the additive term. The final reconstructed SPECT image can then be reconstructed from the proxy using

$$ {\lambda}_j={\sum}_{f=1}^{N_j}{\alpha}_f{k}_{s\  fj} $$

If the PET and SPECT images have the same voxel size and field-of-view (FOV) then the Euclidean distances in the kernel equations are equivalent. The kernel search window size is controlled by the nearest neighbour (NN) parameter; if NN is set to 3 the search window has size 3 × 3 × 3 voxels.

## Experimental procedures

### Phantom study acquisitions

A solution of 3.28 ± 0.09 GBq of ^177^Lu in 1200 ml of water was prepared, where ~48 ml was withdrawn to fill the six spheres in the IEC phantom (diameters 10, 13, 17, 21, 28 and 37 mm), each having a concentration of 2733 ± 85 kBq/ml. The remaining ~1152 ml of the solution was diluted in the background volume of 9787 ± 10 ml so that the sphere-to-background concentration ratio was 8.5:1, with a background concentration of 322 kBq/ml. Aliquots were taken from the spheres and background compartment and were compared to a standard in an auto-gamma counter (2480 Wizard^2^, PerkinElmer, Waltham, MA, USA) so that the radioactivity concentration at scan time in each compartment could be accurately determined. The IEC phantom was scanned on a dual-head SPECT/CT system (Intevo.6, Siemens Healthineers, Hoffman Estates, IL, USA) with medium-energy-low-penetration (MELP) collimators and an acquisition matrix size of 256 × 256. The phantom was scanned for a total of 30 min, where 120 projections were acquired over 360° (3° sampling) using continuous rotation mode, resulting in a 30 s acquisition time per projection. Two energy windows were acquired: ^177^Lu photopeak (187–228 keV) and lower energy scatter window (166–186 keV). The photopeak and lower energy scatter windows were resampled to a matrix size of 128 × 128 to be used in all subsequent SPECT reconstructions. The SPECT data were reconstructed using in-house quantitative SPECT (qSPECT) software using the standard ordered-subset expectation-maximisation (OSEM) algorithm with 4 iterations (it) and 8 subsets (s) using CT-based attenuation and transmission-dependent scatter correction without RM [25, 26]. The data were then reconstructed in STIR using a dual energy window-based scatter correction method [27, 28] with CT-based attenuation correction and additional RM (OSEM + RM). Two STIR OSEM + RM reconstructions were produced: 5 and 40 iterations each with 12 subsets. The qSPECT and STIR OSEM + RM reconstructions had no post-filtering applied. The CT-derived attenuation correction factor maps (“μ-maps”) for both the qSPECT reconstruction and the STIR reconstructions were generated using software developed in-house (IDL, Harris Geospatial, Boulder, CO, USA) [29]. The number of iterations for the OSEM + RM reconstruction was chosen such that the reconstructed result demonstrated a noticeable improvement in recovery of the radionuclide concentration in the three smallest spheres compared to the qSPECT reconstruction (OSEM without RM).

The ^68^Ga IEC phantom study, with an 8.5:1 sphere-to-background ratio, was acquired on a time-of-flight PET/CT (Biograph mCT/64, Siemens, Hoffman Estates, IL, USA) and was scanned for 300 s per bed for two bed positions. The data were reconstructed using the vendor software with 3 iterations and 21 subsets with RM + time-of-flight (TOF) enabled. The reconstructed images were subsequently filtered with a 3D Gaussian function of 5 mm FWHM. The OSEM + RM (40it, 12s) reconstruction of the SPECT data was used to co-register the ^68^Ga PET image to the SPECT frame of reference using a 6 degrees-of-freedom rigid body registration tool (MultiModality, Hermes Medical, Sweden). The SPECTRE reconstruction used the HKEM algorithm implemented in STIR, using the ^68^Ga PET IEC phantom study (resampled and co-registered to the SPECT frame of reference) as the prior for the ^177^Lu SPECTRE reconstruction. The SPECTRE reconstruction used the same number of subsets and iterations as the OSEM + RM (40it, 12s) reconstruction and had no post-filtering to permit direct comparison. The parameters of the HKEM algorithm were selected by manual optimisation based on subjective image quality. The SPECTRE parameters used in the IEC phantom study were: σ_p_ = 2, σ_s_ = 2, σ_dp_ = 5 mm, σ_ds_ = 5 mm, NN = 5. The OSEM + RM and SPECTRE reconstructions using STIR software were executed on a high-performance computer platform (ARTEMIS, University of Sydney, Australia).

### Phantom study analysis

In attempting to examine the effect of finite spatial resolution on partial volume losses in SPECT the main parameters of interest are the quantitative accuracy of the reconstructed values and the amount of statistical uncertainty (“noise”) that this generates. We therefore evaluated the accuracy of the reconstructed radioactivity concentrations, the spatial resolution of the images, and the noise characteristics of the different reconstruction methods. To assess the accuracy, measurements of the target spheres’ contents for each reconstruction were performed using a threshold-based volume-of-interest (VOI) (HybridDisplay, Hermes Medical, Sweden), where the VOI was generated using a variable threshold (%) based on the maximum voxel value in the image. The VOIs that were generated resulted in spherical volumes as close as possible to the known volume of each of the six IEC phantom spheres. Image-based mean and maximum concentration values in each VOI were compared to the known radioactivity concentration measured in the aliquots and were used as a measure to compare each of the SPECT reconstruction methods. The mean and maximum recovery coefficient (RC) was recorded for each VOI, where RC_mean_ and RC_max_ are the mean and maximum activity concentrations in each VOI divided by the known activity concentration. Circular regions of interest (ROIs) were generated in the background compartment so that RC_mean_ could be determined.

Image-based coefficient of variation $$ \left(\raisebox{1ex}{${\sigma}_{\mathrm{VOI}}$}\!\left/ \!\raisebox{-1ex}{${\overline{x}}_{\mathrm{VOI}}$}\right.\times 100\right) $$ (COV%) measurements were derived from the ROIs in the background compartment and were used to assess the noise characteristics of the SPECT and PET reconstructed images. The COV is defined as the standard deviation in the ROI divided by the mean in the ROI. The radioactivity concentration in the IEC phantom background compartment is known, thus, an ideal reconstruction is a trade-off between comparatively small COVs in the background ROIs, indicative of lower variation in voxel values where no variation is expected, and improved quantitative accuracy (better recovery of the known concentration). In the case of increased noise and the presence of Gibbs artefacts, a comparatively smaller COV is a good indication that these undesirable image properties have been minimised. The SPECT image quality measurements RC_mean_, RC_max_ and COV in each VOI/ROI were compared to the same measurements obtained in the ^68^Ga PET IEC phantom image in order to assess the overall quality of the SPECT reconstruction methods compared to PET. Line profiles (LPs) through the centre slice of each sphere were also used as a means of comparing each SPECT reconstruction method.

We also examined the “global” spatial resolution of each reconstructed image using a method previously described [30, 31]. Briefly, starting with a “perfect” PET/SPECT scan based on the segmented CT image of the phantom with appropriate voxel values representing the known radioactivity concentrations for the spheres and background compartment, the volume is iteratively blurred with a 3D Gaussian function of increasing width until the best match between the measured data and the successively blurred image is found using the minimum of the sum of squared differences (MSSD) between the two images. The perfect “ground truth” ^177^Lu IEC image was generated using an IDL program developed in-house, where each compartment of the phantom was assigned an apparent radioactivity concentration determined by the sampled aliquot measurements decay-corrected to the scan time, resulting in a sphere concentration of 2784 kBq/ml, and a background concentration of 317 kBq/ml. This ground truth image was co-registered to the ^177^Lu SPECT reconstructed images, as well as the ^68^Ga PET image (with its own ground truth image concentrations), so that both sets of data were in the same frame of reference. The ground truth image was successively blurred using a 3D Gaussian function with FWHM increments of 0.1 mm, and a difference image between the blurred ground truth image and the SPECT and PET reconstructed images was generated. The approximate spatial resolutions of the SPECT and PET reconstructions were determined as the FWHM of the Gaussian that resulted in the lowest MSSD value. A root mean square error (RMSE) analysis was also performed on the spheres in the PET and SPECT reconstructed images. Using the “ground truth” (GT) images, a squared error (SE) image between this ground truth image and the reconstructed image was generated. The same VOIs as those used to determine RC_mean_ and RC_max_ were propagated to the SE image and the unsigned RMSE was calculated. The results of the RMSE analysis for the PET and SPECT series were normalised (i.e. NRMSE) to the ground truth sphere concentrations to facilitate direct comparison.

### Clinical example with [^64^Cu/^67^Cu]SARTATE

The SPECTRE reconstruction approach was then applied to a clinical theranostic study using [^64^Cu/^67^Cu]MeCOSar-Octreotate (“SARTATE”) (Clarity Pharmaceuticals, Sydney, Australia) in a trial of subjects who were being treated for unresectable, multi-focal meningioma. The subject presented here received 177 MBq of [^64^Cu]SARTATE (^64^Cu t_½_ = 12.7 h, 17% branching ratio) for treatment planning and was imaged using the same PET/CT scanner as the phantom study. The patient was scanned from vertex of skull to mid-thigh at 1, 4 and 24 h post-injection, with a scan time per bed position of 180 s, 180 s and 300 s in the respective scans. Subsequently, images from cycle 1 of ^67^Cu therapy with 5.1 GBq of [^67^Cu]SARTATE (^67^Cu t_½_ = 6.7 days; *E*_γ_ = 185 keV, 49% branching ratio) administered 4 weeks after ^64^Cu imaging were used in the SPECTRE reconstruction. ^67^Cu SPECT imaging was performed using the same dual-head SPECT/CT system as described previously, with medium-energy-low-penetration (MELP) collimators and an acquisition matrix size of 128 × 128. The 4-h time point data were acquired for 8 s per projection at 3 bed positions covering the vertex of skull to mid-thigh. One hundred twenty projections were acquired over 360° (3° sampling) using continuous rotation mode. Two energy windows were acquired: ^67^Cu photopeak (163–199 keV) and a lower energy scatter window (143–162 keV). The 4-h post-injection time points for both the PET and SPECT acquisitions were used in the example presented here. An initial OSEM + RM reconstruction of the SPECT data was used to co-register the 4-h ^64^Cu PET image to the matched time-point ^67^Cu SPECT data, to be used as the guiding modality in the SPECTRE reconstruction.

Quantitative SPECT reconstructions used for direct comparisons were the same as those investigated in the IEC phantom study, namely, a conventional OSEM reconstruction with CT-based attenuation and scatter correction (qSPECT) with no post-reconstruction filtering or RM, the STIR-equivalent OSEM implementation with RM (OSEM + RM) and the SPECTRE reconstruction using the HKEM algorithm with RM, with CT-based attenuation correction and window-based scatter correction. No post-reconstruction filter was applied to the OSEM + RM and SPECTRE reconstructions. The number of iterations and subsets for the qSPECT, OSEM + RM and SPECTRE reconstructions were the same as those used in the phantom study, i.e. 4it8s, 40it12s and 40it12s, respectively. The SPECTRE parameters used in the clinical example were σ_p_ = 1, σ_s_ = 1, σ_dp_ = 5 mm, σ_ds_ = 5 mm, NN = 3. As in the phantom example, these parameters were chosen based on subjective image quality. Quantitative images with voxel units of kBq/ml were generated for each of the SPECT reconstructions and were co-registered to the matched time-point PET image. VOIs were generated on the PET image using a threshold (% of maximum value in the image) and were propagated to each of the SPECT reconstructed images. The SUV_mean_ and SUV_max_ in each VOI for all reconstructed images was calculated and subsequently used in our analysis.

## Results

### Phantom study

**Fig. 1:**
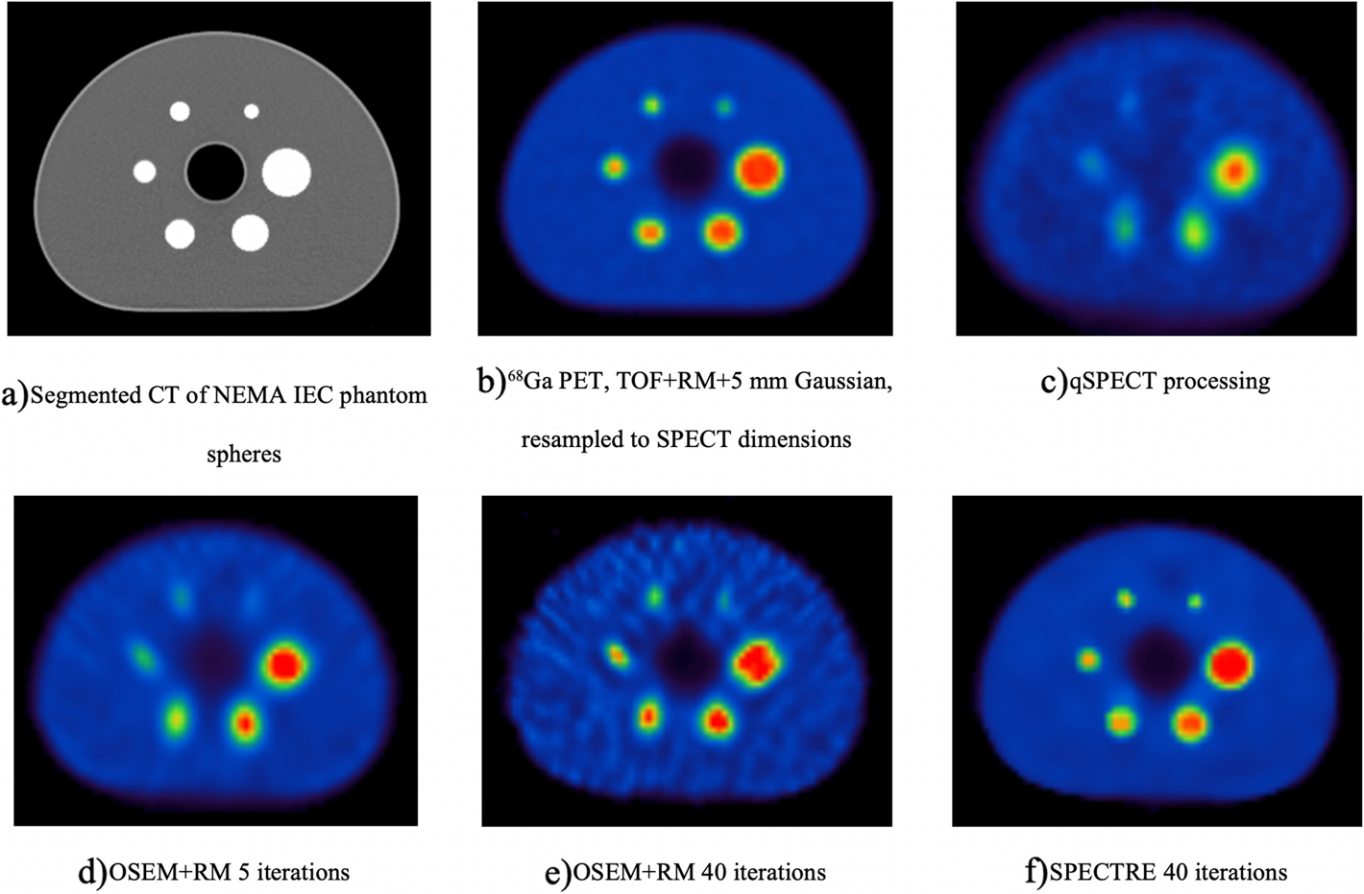
Transverse centre slice of IEC image quality phantom for each reconstruction method. **a** CT image (segmented spheres), **b** ^68^Ga PET, TOF + RM + 5 mm Gaussian, resampled to SPECT dimensions and used as prior image used in the SPECTRE reconstruction. **c** Our in-house routine qSPECT-OSEM reconstruction (4 iterations and 8 subsets), **d** OSEM + RM reconstruction (5 iterations and 12 subsets), **e** OSEM + RM reconstruction (40 iterations and 12 subsets), and **f** SPECTRE with RM [HKEM parameters: σ_p_ = 2, σ_s_ = 2, σ_dp_ = 5, σ_ds_ = 5, NN = 5] (40 iterations and 12 subsets)

Figure 1 shows transverse slices of the PET and SPECT IEC phantom images with the same windowing applied to each reconstruction. The OSEM + RM − 40 iterations with 12 subsets (40it12s) reconstruction is labelled as “OSEM+RM” and is the study used in all subsequent analyses.

**Fig. 2:**
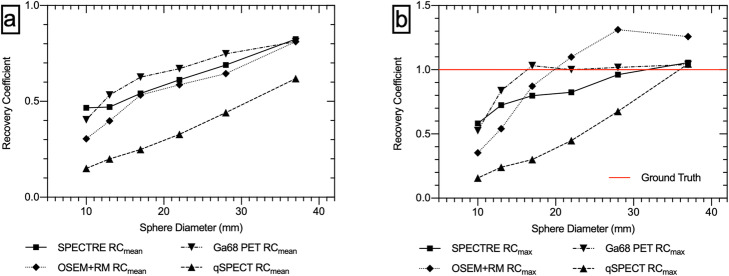
**a** Recovery coefficient of the mean concentration in each sphere. **b** Recovery coefficient of the max value in each sphere

The recovery coefficients RC_mean_ and RC_max_ versus sphere diameter for the ^68^Ga PET and ^177^Lu SPECT reconstructions are shown in Fig. 2.

The mean recovery (RC_mean_) in the SPECTRE reconstruction is higher than the OSEM + RM equivalent in all six spheres. The qSPECT reconstruction has a RC_mean_ of 0.62 in the largest sphere and 0.15 in the smallest sphere, compared to SPECTRE which has a RC_mean_ of 0.82 in the largest sphere and 0.47 in the smallest sphere, showing an improvement in recovery in the largest and smallest sphere by a factor of 30% and > 300% respectively. The maximum recovered voxel values (RC_max_) in the four largest spheres of the ^68^Ga PET series achieves full recovery of the expected radioactivity concentration, whereas the SPECTRE reconstruction only sees full recovery in the two largest spheres. The OSEM + RM reconstruction overestimates the expected concentration in the three largest spheres. Full recovery of the expected concentration in the qSPECT series is only recovered in the largest sphere (RC_max_ = 1.05). Table 1 shows the NRMSE in the sphere VOIs and the COV% and RC_mean_ in the background ROIs for each of the PET and SPECT reconstructions.

**Table 1:** NRMSE in the sphere VOIs and COV% and RC_mean_ in the background ROIs for ^68^Ga PET, qSPECT, OSEM + RM and SPECTRE. BKG is the background compartment of the phantom

Sphere Diameter (mm)	^68^Ga PET 3it21s NRMSE	qSPECT 4it8s NRMSE	OSEM + RM 40it12s NRMSE	SPECTRE 40it12s NRMSE
37	**0.25**	**0.41**	**0.30**	**0.26**
28	**0.31**	**0.57**	**0.42**	**0.35**
22	**0.37**	**0.67**	**0.45**	**0.41**
17	**0.41**	**0.75**	**0.49**	**0.49**
13	**0.49**	**0.80**	**0.61**	**0.54**
10	**0.60**	**0.85**	**0.70**	**0.54**
BKG RC_mean_	**0.99**	**0.95**	**1.04**	**1.03**
BKG COV%	**4.1**	**8.7**	**20.0**	**4.3**

**Fig. 3:**
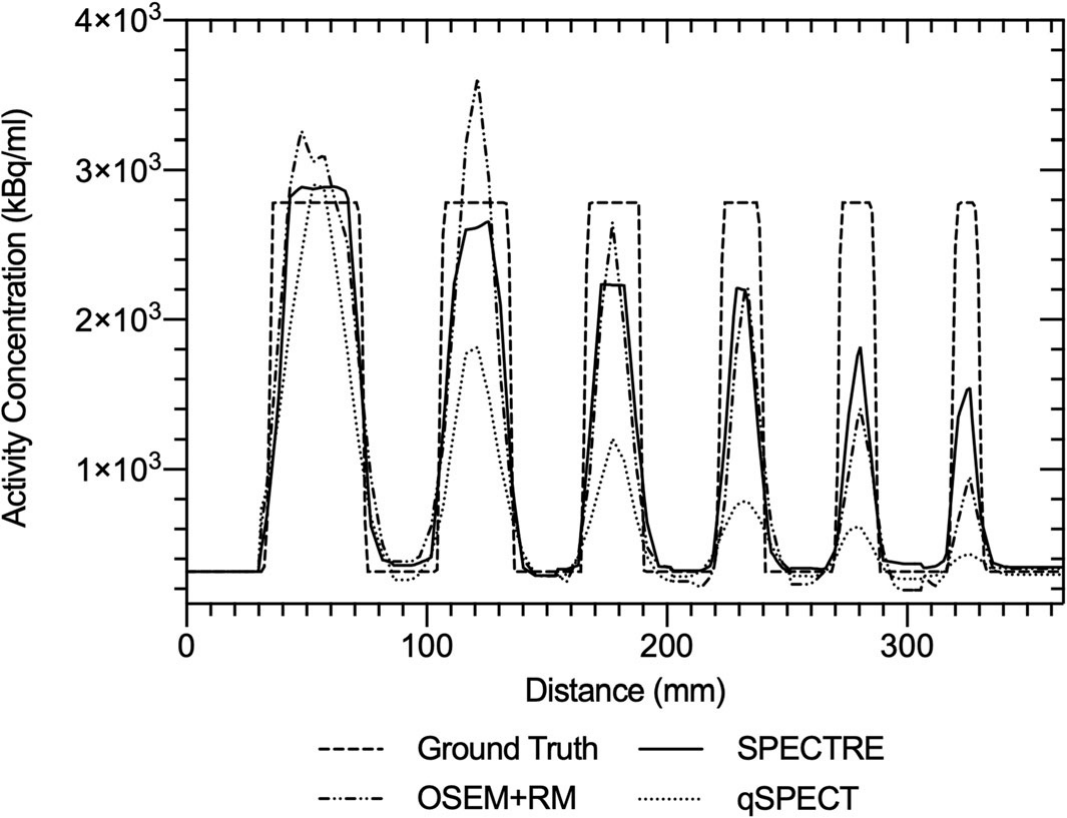
Line profiles through the centre of each sphere for each SPECT reconstruction method, qSPECT(4it8s), OSEM + RM(40it12s) and SPECTRE(40it12s), compared to ground truth

Line profiles drawn through the centre slice of each sphere for each SPECT reconstruction are shown in Fig. 3.

The global spatial resolution analysis of the ^68^Ga PET image found that the MSSD occurred when using a 3D Gaussian blurring kernel with a FWHM of 7.5 mm, suggesting that this is the approximate resolution of the PET reconstructed image using our standard clinical reconstruction parameters. The MSSD analysis for each SPECT reconstruction suggests that the FWHM spatial resolution of the ^177^Lu qSPECT reconstruction was approx. 18.2 mm, for OSEM + RM it was 10 mm and for SPECTRE it was 8.8 mm.

### Clinical patient data—^64^Cu/^67^Cu-SARTATE

**Fig. 4:**
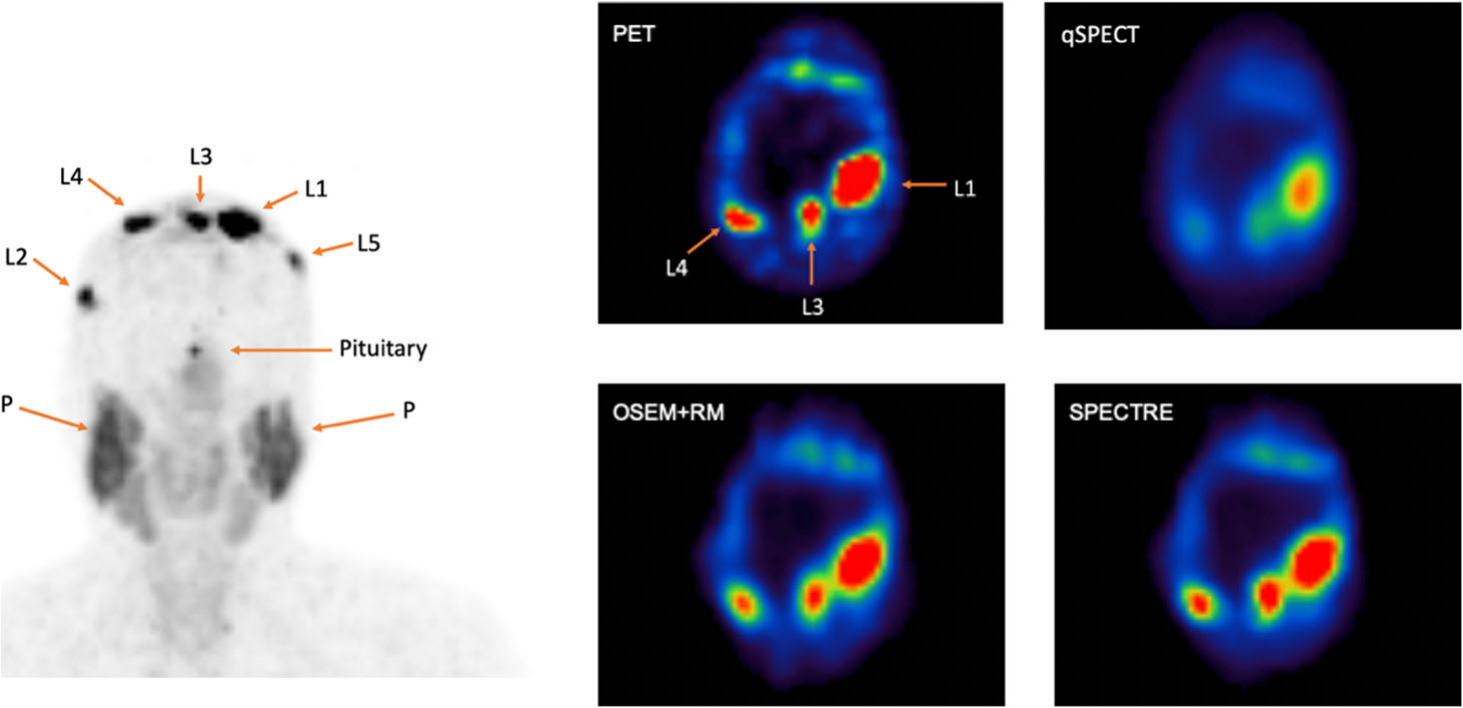
Left: Maximum intensity projection (MIP) of 4-h ^64^Cu-PET head with VOIs labelled. Upper row centre: ^64^Cu-PET image (OSEM + RM + 5 mm Gaussian filter): transverse slice showing L1, L3 and L4. The same transverse slice, ^67^Cu-SPECT; top right: qSPECT OSEM (4 iterations and 8 subsets), lower row centre: OSEM + RM (40 iterations and 12 subsets), bottom right: SPECTRE with resolution modelling. Abbreviations: L–lesion; P–parotids

Each SPECT reconstruction method using the ^67^Cu data was compared to the same time-point ^64^Cu PET image. Five lesions in the PET image were identified and contoured, along with the pituitary and the parotid glands, both of which demonstrate increased uptake indicative of the presence of somatostatin receptors. Figure 4 shows a maximum intensity projection (MIP) of the 4-h ^64^Cu PET reconstruction with each VOI labelled (left side of the figure). Next to this, the same transverse slices for all reconstructions are displayed showing lesions 1, 3 and 4 in a single section through the skull. The same windowing has been applied to each SPECT reconstructed image.

It is evident in the qSPECT image that lesions 1, 3 and 4 have poor recovery, as would be expected, compared to PET and both SPECT (OSEM + RM and SPECTRE) reconstructions. The PVE is best illustrated in lesion 4 on the qSPECT reconstructed image; under identical windowing the uptake seen in the VOI is very faint compared to the other reconstructions. The corresponding SUV_mean_ and SUV_max_ for each VOI are shown in Table 2.

**Table 2:** Mean and max SUVs for VOIs within the head of the test patient

VOI	Vol (ml)	SUV_mean_				SUV_max_			
		PET	qSPECT	OSEM + RM	SPECTRE	PET	qSPECT	OSEM + RM	SPECTRE
L1	**3.8**	**14.0**	**9.4**	**20.7**	**19.6**	**22.4**	**12.6**	**38.7**	**36.6**
L2	**1.3**	**5.3**	**2.4**	**7.7**	**7.4**	**9.6**	**3.0**	**13.0**	**12.1**
L3	**1.3**	**9.5**	**4.5**	**11.5**	**12.2**	**16.5**	**5.5**	**17.0**	**19.9**
L4	**1.0**	**7.7**	**3.2**	**10.5**	**10.5**	**11.9**	**3.9**	**15.2**	**15.5**
L5	**1.1**	**3.9**	**2.0**	**4.5**	**4.4**	**7.6**	**2.3**	**6.8**	**6.7**
Pituitary	**0.6**	**4.2**	**0.7**	**2.4**	**2.8**	**7.1**	**0.8**	**3.0**	**3.6**
Parotid–right	**23.1**	**4.6**	**4.5**	**6.6**	**6.5**	**6.7**	**7.6**	**13.6**	**13.2**
Parotid–left	**22.1**	**4.4**	**4.4**	**6.4**	**6.6**	**6.6**	**6.4**	**10.6**	**9.8**

L denotes lesion

Table 2 shows that the standard OSEM ^67^Cu SPECT reconstruction without RM (qSPECT) has significantly lower recovery in small VOIs compared to its theranostic ^64^Cu PET counterpart. The qSPECT ^67^Cu SUV_mean_ in each lesion is, on average, half that of the ^64^Cu PET SUV_mean_ estimate. The COV% in the parotids is 16.3%, 25.5%, 31.5% and 26.9% for the PET, qSPECT, OSEM + RM and SPECTRE reconstructions, respectively.

## Discussion

The field of theranostics in nuclear medicine has enjoyed a great deal of expansion over the past decade. Importantly, this is translating into improved outcomes for patients [32]. However, many of the protocols that are being used to treat patients with various malignancies are not optimised or tailored to the individual—so-called “personalised medicine”. One of the reasons for this is the lack of knowledge between the radiation dose delivered to a target lesion and the response that is elicited. It is fortuitous that many of the therapeutic radionuclides using β^-^ emission to deliver the radiation dose also emit photons which can be imaged with conventional gamma cameras. The two radionuclides used in this study both emit photons around 200 keV. The spatial resolution of the gamma camera operating with photons of this energy is poor, measuring around 20 mm FWHM. As many of the metastatic lesions and malignant nodal disease are of this size or smaller it means that we are unable to accurately estimate the amount of radiation delivered to the tissues without addressing the issue of partial volume correction. This, in turn, hampers our ability to better understand the radiobiology of radionuclide therapy, especially in the metastatic setting, and hence lead to more personalised treatments.

### Phantom study

In our phantom study using a combination of ^68^Ga PET/^177^Lu SPECT, the SPECT reconstructions that incorporated RM showed improved recovery of the radioactivity concentration in all six of the spheres. The SPECTRE reconstruction showed better recovery of the mean activity concentration in all six of the IEC phantom spheres compared to OSEM + RM for the same number of iterations, with a more noticeable improvement in recovery in the two smallest spheres. Recovery of the concentration in the background (BKG RC_mean_), that is quantitative accuracy, was similar for all PET and SPECT reconstructions and was within ± 5% of the known background concentration. The SPECTRE reconstruction (for the chosen HKEM parameters) and the ^68^Ga PET image have similar noise properties in the background compartment, with a COV of 4.3% and 4.1% respectively, compared to the OSEM + RM reconstruction which has a COV of 20.0%, indicative of higher noise in the background. The OSEM + RM (40it12s) reconstruction has a “blobby” noise texture in the background, a result likely due to the reconstruction algorithm fitting the noise in the data too closely, resulting in a modified noise texture that has supressed high-frequency noise features at the cost of amplifying mid-frequency noise correlation with a larger number of iterations [5, 33]. This structured noise artefact has scarcely been discussed in the literature, however, brief mentions of midfrequency noise correlation due to resolution recovery methods can be found in [34–36].

Figure 2b shows that the OSEM + RM (40 iterations) reconstruction overestimates the expected radioactivity concentration in the three largest spheres. This can be attributed to Gibbs artefacts often leading to overestimation of the true concentration with the “peaks” merging together and resulting in even greater overestimates [11]. Figure 3 shows a comparison of line profiles through the centre of all IEC phantom spheres for the qSPECT, OSEM + RM and SPECTRE reconstructions. Gibbs-type artefacts can be seen in the OSEM + RM profile. The profile through the largest sphere shows a “dip” in the centre, with high activity at the edges (ringing), and the profile through the second largest sphere shows a large overshoot of the expected concentration, indicative of Gibbs-type artefacts merging together. A large overestimation of the true sphere concentration and a blobby noise texture in the background compartment are features only present in the OSEM+RM reconstructed image.

We have previously reported that a ^68^Ga point source placed in the centre of the FOV of our PET/CT system has a transverse spatial resolution of approx. 5.8 mm FWHM [3]. The estimated global spatial resolution from the MSSD analysis of the ^68^Ga PET IEC acquisition was 7.5 mm FWHM, which is consistent with our previous report of the intrinsic spatial resolution of ^68^Ga PET imaging: intrinsic reconstructed resolution of 5.8 mm FWHM with a post-reconstruction Gaussian filter of 5 mm FWHM results in a resolution of approx. $\sqrt{5.8^2+{5.0}^2}$ = 7.7 mm FWHM. The spatial resolution of the ^177^Lu IEC qSPECT reconstructed image was found to be 18.2 mm FWHM compared to the estimated SPECTRE resolution of 8.8 mm FWHM, suggesting an improvement in resolution by a factor of ~2. Improving spatial resolution by a factor of two leads to an expected improvement of volume resolution of a factor of 8 (i.e. 2^3^). For comparison, the OSEM+RM reconstructed SPECT image was found to have a global resolution of 10 mm, demonstrating that conventional PVC methods such as RM do well in recovering radioactivity concentrations and improving resolution, but noise amplification, introduction of Gibbs artefacts, and the overestimation of the true radioactivity concentration in the spheres support the fact that more sophisticated approaches to partial volume correction are needed. The NRMSE results reported in Table 1 further demonstrate that the SPECTRE reconstruction recovered the concentration in the spheres more accurately (closer to true value) than the qSPECT and OSEM + RM reconstructions; the SPECTRE reconstruction had a lower NRMSE in 5 out of the 6 spheres, with only the NRMSE in the 17-mm-diameter sphere being the same for both SPECTRE and OSEM + RM. Overall, the SPECTRE reconstruction showed an improvement in the spatial resolution using medium energy collimators and a reduction in noise amplification and structured noise artefacts compared to the OSEM + RM reconstruction. The SPECTRE reconstruction of the ^177^Lu IEC phantom data demonstrates that a PET image can be used to guide the SPECT reconstruction, producing images with comparable resolution, and hence recovery of true radiopharmaceutical uptake, to PET.

### Clinical patient data: ^64^Cu/^67^Cu-SARTATE

The reconstruction methods that address the collimator-detector response resulted in a higher SUV_mean_ and SUV_max_ in small structures, where the proposed SPECTRE method saw an average increase by a factor of approx. 2.7 in the mean radioactivity concentration in small lesions when compared to the standard qSPECT reconstruction. In Fig. 4, one particular lesion (L4) in the PET image, which is very faint in the qSPECT reconstruction, shows that the OSEM + RM and SPECTRE reconstructions both saw an increase in SUV_mean_ by a factor of 3.3. All VOIs in the SPECTRE and OSEM + RM reconstructions have a higher SUV_mean_ and SUV_max_ than the respective PET measurement, except for the pituitary gland which is significantly higher in PET. This may be attributed to the different physiological conditions between diagnostic imaging and therapy where an amino acid infusion is administered for nephroprotection purposes during PRRT. The amino acid infusion could have a pharmacokinetic effect on the pituitary uptake and retention, causing the radioligand to washout more quickly. The co-administration of amino acids during ^67^Cu therapy makes it difficult to directly compare uptake in the PET and SPECT images as the biodistribution may be affected. The parotids, being the largest volume investigated in this study, are suitable for comparing the noise characteristics in each of the reconstructed images. For this VOI, the SPECTRE reconstruction had similar noise properties to the qSPECT reconstruction, which was very blurred and hence relatively low in noise level. In contrast, the OSEM + RM reconstruction had a significantly higher COV%, indicative of a higher level of noise.

### General discussion

Our investigations into use of the HKEM algorithm for theranostic PET-guided SPECT image reconstruction have identified a number of issues that need to be considered and investigated further. The HKEM algorithm was originally implemented for reconstructing emission data (PET) using anatomical image data (CT or MRI) as the guiding modality and thus has not yet been optimised for the use of functional data as the guiding modality. Further investigation into how to best implement functional data as the prior needs to be explored. Additionally, accurate spatial co-registration between the PET and SPECT data is a requirement and is affected by issues such as different bed shapes between the PET and SPECT cameras, the position of the subject’s arms (i.e. above their head or by their side) in each modality, and the presence of physiological motion or variability (e.g. diaphragmatic motion, degree of urinary bladder filling, peristalsis and general movement of the small and large bowel). As we chose to use the head for our clinical study, which is relatively immune to spatial variations in features, the dependence of the SPECTRE algorithm on these spatial misalignments has not been tested as yet and will be the subject of future work.

The superior noise properties of the SPECTRE reconstructions seen in this work suggests the possibility that this approach to SPECT image reconstruction will be useful in the reconstruction of low-count SPECT data and this will also be the subject of future work. Optimisation of the SPECTRE reconstruction approach in a theranostic setting will likely involve joint optimisation of the PET image reconstruction, as the guiding modality, combined with a range of HKEM parameters in order to achieve an optimal SPECTRE reconstruction result. Robustness to accurate registration and sensitivity to the differences in the PET and SPECT pharmacokinetics need to be considered.

## Conclusion

SPECT imaging plays an important role in monitoring organ and lesion absorbed dose but is severely limited by its poor spatial resolution, particularly for medium energy gamma photon imaging. Mitigation of PVEs in order to obtain accurate lesion and normal organ dosimetry is a much-needed step towards personalised RNT, where individualised doses, patient-specific treatment planning, and investigation into radiobiological effects and response to treatment are all directly linked to our ability to make accurate image-based dosimetry measurements. We have demonstrated that the theranostic paradigm can be exploited in SPECT image reconstruction to improve the poor spatial resolution of gamma camera imaging of therapeutic radionuclides when the corresponding PET image is used as guiding information incorporated into the reconstruction process. This novel approach to SPECT image reconstruction, using matched pairs of diagnostic (PET) and therapeutic (SPECT) imaging, is a promising method for improving SPECT image quality and thus our ability to accurately monitor treatment, leading to better quantification of the dose delivered to small structures such as nodal and metastatic disease and target lesions. The SPECTRE approach to image reconstruction shows clear improvements over conventional reconstructions incorporating resolution modelling due to its better noise characteristics and lack of Gibbs and other resolution modelling artefacts. Further investigation and optimisation of the algorithm parameters is needed and should be investigated for a number of theranostic oncological applications that use radionuclides for therapy.

## Data Availability

We do not have permission to make the data available.

## Abbreviations

BKG: Background
COV: Coefficient of variation
CT: Computed tomography
FOV: Field of view
FWHM: Full-width at half maximum
HKEM: Hybrid kernelised expectation maximisation
KEM: Kernelised expectation maximisation
LP: Line profile
MRI: Magnetic resonance imaging
MSSD: Minimum sum of squared differences
NN: Nearest neighbour
NRMSE: Normalised root mean square error
OSEM: Ordered subset expectation maximisation
PET: Positron emission tomography
PVC: Partial volume correction
PVE: Partial volume effect
RC: Recovery coefficient
RM: Resolution modelling
RMSE: Root mean square error
RNT: Radionuclide therapy
ROI: Region of interest
SPECT: Single photon emission computed tomography
SPECTRE: Single photon emission computed theranostic reconstruction
STIR: Software for tomographic image reconstruction
SUV: Standard uptake value
TIR: Theranostic image reconstruction
TOF: Time of flight
VOI: Volume of interest

## Footnotes

1 The term “theranostic” is attributed to the American John Funkhouser in a press release from the company *Cardiovascular Diagnostics* in August 1998 [1].

2 Available at http://stir.sourceforge.net
